# Impact of Tumour Hypoxia on Evofosfamide Sensitivity in Head and Neck Squamous Cell Carcinoma Patient-Derived Xenograft Models

**DOI:** 10.3390/cells8070717

**Published:** 2019-07-13

**Authors:** Julia K. Harms, Tet-Woo Lee, Tao Wang, Amy Lai, Dennis Kee, John M. Chaplin, Nick P. McIvor, Francis W. Hunter, Andrew M. J. Macann, William R. Wilson, Stephen M.F. Jamieson

**Affiliations:** 1 Auckland Cancer Society Research Centre, University of Auckland, Auckland 1023, New Zealand; 2 Maurice Wilkins Centre for Molecular Biodiscovery, University of Auckland, Auckland 1010, New Zealand; 3 Department of Pharmacology and Clinical Pharmacology, University of Auckland, Auckland 1023, New Zealand; 4 LabPLUS, Auckland City Hospital, Auckland 1023, New Zealand; 5 Department of Otolaryngology–Head and Neck Surgery, Auckland City Hospital, Auckland 1023, New Zealand; 6 Department of Radiation Oncology, Auckland City Hospital, Auckland 1023, New Zealand

**Keywords:** tumour hypoxia, head and neck squamous cell carcinoma (HNSCC), evofosfamide, patient-derived xenograft (PDX), pimonidazole, POR, MKI67, SLFN11

## Abstract

Tumour hypoxia is a marker of poor prognosis and failure of chemoradiotherapy in head and neck squamous cell carcinoma (HNSCC), providing a strategy for therapeutic intervention in this setting. To evaluate the utility of the hypoxia-activated prodrug evofosfamide (TH-302) in HNSCC, we established ten early passage patient-derived xenograft (PDX) models of HNSCC that were characterised by their histopathology, hypoxia status, gene expression, and sensitivity to evofosfamide. All PDX models closely resembled the histology of the patient tumours they were derived from. Pimonidazole-positive tumour hypoxic fractions ranged from 1.7–7.9% in line with reported HNSCC clinical values, while mRNA expression of the Toustrup hypoxia gene signature showed close correlations between PDX and matched patient tumours, together suggesting the PDX models may accurately model clinical tumour hypoxia. Evofosfamide as a single agent (50 mg/kg IP, qd × 5 for three weeks) demonstrated antitumour efficacy that was variable across the PDX models, ranging from complete regressions in one p16-positive PDX model to lack of significant activity in the three most resistant models. Despite all PDX models showing evidence of tumour hypoxia, and hypoxia being essential for activation of evofosfamide, the antitumour activity of evofosfamide only weakly correlated with tumour hypoxia status determined by pimonidazole immunohistochemistry. Other candidate evofosfamide sensitivity genes—*MKI67*, *POR*, and *SLFN11*—did not strongly influence evofosfamide sensitivity in univariate analyses, although a weak significant relationship with *MKI67* was observed, while *SLFN11* expression was lost in PDX tumours. Overall, these data confirm that evofosfamide has antitumour activity in clinically-relevant PDX tumour models of HNSCC and support further clinical evaluation of this drug in HNSCC patients. Further research is required to identify those factors that, alongside hypoxia, can influence sensitivity to evofosfamide and could act as predictive biomarkers to support its use in precision medicine therapy of HNSCC.

## 1. Introduction

Hypoxia, a deficiency in oxygen in tissues, arises selectively in tumours through inefficient blood flow and oxygen delivery as a result of the irregular and dysfunctional vasculature that forms as tumours grow [1,2,3], as well as through oxygen consumption in tumours [4,5] and adaptations to survive under severe hypoxia [6]. Tumour hypoxia promotes cancer progression through hypoxia-induced factor (HIF)-dependent signalling [7], the unfolded protein response [8] and epigenetic regulation [9,10] leading to cell survival, invasiveness and metastasis [11]. Hypoxic cells are resistant to radiotherapy, which requires oxygen to induce DNA double-strand breaks [1] and to many chemotherapy drugs through multiple mechanisms including cell cycle arrest [12], protection from apoptosis [13,14], and limited penetration into hypoxic regions of tumours [15,16]. Hypoxia can also limit the activity of immunotherapy drugs by activating immune checkpoints [17], recruiting regulatory T cells [18], and suppressing T-cell function [19].

The evidence supporting hypoxia as an oncology target is strongest in head and neck squamous cell carcinoma (HNSCC), where hypoxia is a marker of poor prognosis and failure of chemoradiotherapy [20,21,22,23,24]. HNSCC comprises >90% of primary tumours within the head and neck region [25], which collectively are the 7th most common cancer presentations and cause of cancer-related deaths worldwide [26]. Major risk factors for HNSCC include human papilloma virus (HPV) infection [27] and smoking and alcohol use [28,29]. HPV-positive disease generally has a favourable prognosis. However, for HPV-negative disease, patient survival has not markedly improved in recent decades and is limited by locoregional failure, metastases, and resistance to therapy.

Multiple strategies have been developed to target tumour hypoxia, including the use of hypoxia-activated prodrugs (HAPs) that are designed to selectively target tumours over healthy tissues [11]. HAPs are activated by enzymatic reduction in the absence of oxygen to selectively release cytotoxins [30,31,32,33,34] or molecularly-targeted agents [35,36,37] within hypoxic regions of tumours that can potentially diffuse out to act on neighbouring oxygenated tumour cells to provide a bystander effect [38]. Evofosfamide (TH-302) is the most clinically-evaluated HAP and has demonstrated evidence of antitumour activity in preclinical models [39,40,41,42,43,44,45], and in patients [46,47,48], including in HNSCC [49].

Hypoxia is a well-characterised determinant of sensitivity for HAPs [11,50]. Evofosfamide requires the presence of hypoxia to be converted to its active effectors: the potent DNA-crosslinker bromo-*iso*-phosphoramide mustard (Br-IPM) and its chloro-displacement product chloro-*iso*-phosphoramide mustard [51,52], as under aerobic conditions the initial nitro radical intermediate in this reduction pathway is rapidly oxidised back to the inactive prodrug. Additional markers of sensitivity to evofosfamide have been proposed. These include: POR (NADPH:cytochrome P450 oxidoreductase), which has been reported to catalyse the one-electron reduction of evofosfamide selectively under hypoxia to form Br-IPM [51,52,53]; proliferation markers, since a proliferation metagene can predict evofosfamide sensitivity in HNSCC cell lines in vitro [49] and DNA damage response and mitochondrial electron transport genes (e.g., *SLFN11*, *YME1L1*), identified as evofosfamide sensitivity genes through functional genomic screens [54].

We have previously demonstrated antitumour activity of evofosfamide in a small number of cell line xenograft and patient-derived xenograft (PDX) models of HNSCC in combination with radiotherapy and to a lesser extent as a single agent [49]. Here we establish additional PDX models of HNSCC and evaluate them for their hypoxia status and evofosfamide antitumour efficacy to determine the extent to which tumour hypoxia controls sensitivity to evofosfamide in HNSCC. Furthermore, we evaluate the expression of candidate evofosfamide sensitivity genes, *POR*, *SLFN11* and the proliferation marker and metagene constituent *MKI67* for their impact on evofosfamide sensitivity on our HNSCC PDX models.

## 2. Materials and Methods

### 2.1. Animals

Six to eight-week-old female NOD scid gamma (NSG; NOD.Cg-*Prkdc^scid^ Il2rγ^tm1Wjl^*/SzJ; Jackson Laboratories), NOD scid (NOD.CB17- *Prkdc^scid^*/NCrCrl; Charles River) or NIH-III (NIH-*Lyst^bg-J^ Foxn1^nu^ Btk^xid^*; Charles River) mice were used as hosts for the generation of PDX models. All animals had ad libitum access to food and water in microisolator cages and were maintained on a 12 h light/dark cycle. Animal health and welfare was monitored regularly with animals culled if their condition deteriorated or if they lost in excess of 20% of their pre-manipulation bodyweight. Tumour volume was recorded by electronic callipers using the formula π/6 × width × length^2^. All animal experiments followed procedures approved by the University of Auckland Animal Ethics Committee (approvals: #001190 and #001781).

### 2.2. PDX Models

The collection of patient tumour specimens for PDX engraftment was approved by the New Zealand Health and Disability Ethics Committee (approval: 14/NTB/122) and the Auckland District Health Board. Tumour specimens from consenting patients undergoing surgery at Auckland City Hospital were histologically confirmed to be HNSCC via tumour biopsy with HPV status inferred by p16 immunohistochemistry, as described previously [55]. For PDX model generation, p16-negative tumours were preferred. Tumours were collected in Hanks Balanced Salt Solution (Thermo Fisher Scientific, Auckland, New Zealand) supplemented with 10% foetal calf serum (Moregate Biotech, Hamilton, New Zealand) and 100 units/mL penicillin/100 µg/mL streptomycin (Thermo Fisher Scientific) and cut into small fragments (<20 mm^3^) for immediate engraftment into mice. NSG or NOD scid mice were anaesthetised with 100 mg/kg ketamine (Ceva Animal Health, Glenorie, New South Wales, Australia) and 10 mg/kg xylazine (Phoenix Pharm, Auckland, New Zealand) IP and subcutaneously engrafted with six tumour fragments immersed in matrigel (Corning Inc., New York, NY, USA) through 3–5 mm incisions on bilateral flanks (three fragments per flank). Once these first-passage (P1) tumours reached approx. 1500 mm^3^, they were collected and fragmented for further engraftment or for cryopreservation in the collection media described above supplemented with 10% DMSO (Sigma-Aldrich, Auckland, New Zealand). Second generation (P2) PDX tumours were generated by subcutaneous bilateral engraftment of fresh or thawed P1 fragments into an additional cohort of NSG or NOD scid mice. The P2 tumours were grown to approx. 1500 mm^3^ at which point the mice were treated IP with 60 mg/kg pimonidazole and the tumours were removed 2 h later. The P2 tumours were cut into small fragments for engraftment into NOD scid or NIH-III mice to generate P3 PDX tumours for evaluation of evofosfamide anticancer efficacy. Additionally, a whole tissue slice (approx. 1–2 mm thick) was collected and placed in 10% neutral buffered formalin for 24–48 h. The remainder of the tumour was snap frozen in liquid nitrogen. For antitumour efficacy, NOD scid mice bearing P3 PDX tumours were randomised to receive control vehicle (saline) or 50 mg/kg evofosfamide (provided by Threshold Pharmaceuticals, Inc., San Francisco, CA, USA) by IP injection qd × 5 for three weeks once tumours reached approx. 200 mm^3^. Tumours were measured two to three times a week by electronic callipers until they reached endpoint (tumour volume = 2000 mm^3^).

### 2.3. Pimonidazole Immunostaining and Histology

Clinical tumour specimens and P2 PDX tumours were fixed in 10% neutral buffered formalin for 24–48 h, prior to long-term storage in 70% ethanol. Formalin-fixed tumour fragments were embedded in paraffin wax, cut on a microtome into 10 µM sections and mounted onto glass slides. The slides were deparaffinised and rehydrated by heating at 58 °C for 60 min and rinsing in xylene, ethanol and distilled water. Sections were stained with haematoxylin and eosin (Sigma-Aldrich) and visualised using a Zeiss Axiostar microscope and AxioVision 4.9.1 software (Carl Zeiss Microscopy, Oberkochen, Germany).

Slides with P2 PDX tumour sections from mice treated with pimonidazole were submerged in 10 mM citrate buffer (pH = 6.0) and placed in an Antigen Retriever 2100 pressure cooker (Aptum Biologics, Southampton, UK) for 2.5 h, followed by washing in Tris-buffered saline (TBS) and TBS with 0.1% Tween-20 (TBST). Sections were blocked for 1 h at 4 °C in 10% normal goat serum in TBST and stained overnight at 4 °C with pimonidazole-FITC antibody (mouse monoclonal antibody 4.3 11.3, NPI Inc., Burlington, MA, USA) diluted 1:50 in 5% normal goat serum in TBST. Sections were rinsed in TBST and counterstained with the nuclear marker Hoechst 33258 (Thermo Fisher Scientific). After further washing in TBST and TBS, slides were mounted with coverslips and imaged using a Zeiss Axio Imager Z2 microscope with a VSlide scanner (MetaSystems, Altlussheim, Germany). Two- to six-hundred images were taken at 20x magnification at 365 nm (Hoechst 33258) and 470 nm (FITC) using Metafer software 3.12.9 (MetaSystems) and stitched together using VSlide software 1.1.121 (MetaSystems) to create an image of a complete tumour slice. The pimonidazole-positive fraction was quantitated using ImageJ (v1.52e) by determining the immunostained area as a fraction of the viable tumour area, where necrotic regions were excluded. Necrosis was identified as a lack of Hoechst 33258 staining and was confirmed by histopathological examination of slides following de-coverslipping and staining for haematoxylin and eosin.

### 2.4. Gene Expression

Snap-frozen tumour tissues were pulverised into powder using a BioPulveriser (Biospec Products Inc., Bartlesville, OK, USA) under liquid nitrogen. RNA was extracted from the powder using the Aurum Total RNA Fatty and Fibrous Tissue Kit and PureZOL RNA isolation agent (Bio-Rad Laboratories Inc., Auckland, New Zealand), according to the manufacturer instructions. RNA sample quality was assessed by Nanodrop ND-1000 (Thermo Fisher Scientific) and quantitation by Qubit BR RNA kit on a Qubit 3.0 fluorometer (Thermo Fisher Scientific). The mRNA expression of 18 human genes was assessed alongside three housekeeping genes (*RPS13*, *RPLP0* and *OAZ1* [56]) by NanoString PlexSet assay on an nCounter FLEX (NanoString Technologies Inc., Seattle, WA, USA). Oligonucleotide probe pairs for each gene (Table S1) were purchased from Integrated DNA Technologies (Coralville, IA, USA). Optimal sample input for NanoString analysis was determined using an nCounter PlexSet Titration Kit. Samples were hybridised to pooled probe pairs in the presence of Master Mix, and run on an nCounter FLEX alongside a reference calibration sample as per the manufacturer’s instructions. Quality control was confirmed by geNorm analysis and count normalisation carried out on nSolver 4.0 software (NanoString Technologies Inc.) using the geometric mean of the housekeeping genes.

### 2.5. Toustrup Hypoxia Gene Signature

The normalised counts from the NanoString PlexSet assay were used to determine the median expression across all PDX tumours for each of the 15 Toustrup hypoxia signature genes. Each PDX tumour sample was attributed scores of +1 for expression above the median and −1 for expression below the median for each of the 15 genes. These individual gene scores were summed to give a Toustrup hypoxia score between 15 (most hypoxic) and −15 (least hypoxic) for each tumour, an approach analogous to that reported recently for the Buffa hypoxia signature [57,58].

### 2.6. Statistics

Tumour growth inhibition by evofosfamide was determined in each model by comparing average tumour volume in treatment mice vs. controls at the completion of treatment, or earlier if animals reached endpoint, by Student’s T-test. The time taken for tumours to quadruple in size (relative tumour volume × 4; RTV^4^) was used as a survival endpoint, with differences in the median value between controls and treatment assessed by log-rank test. Pearson correlation coefficients were determined for correlation analyses. All statistical analyses were carried out using GraphPad Prism v8.00 (San Diego, CA, USA).

## 3. Results

### 3.1. Establishment of PDX Models of HNSCC

**Figure 1:**
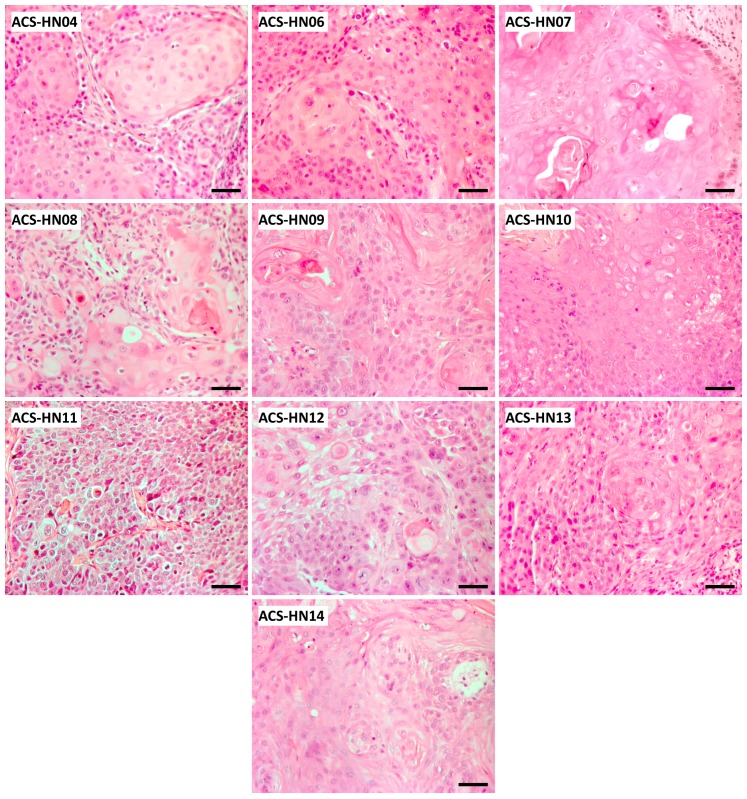
Histopathology of ten PDX models at second passage (P2). Scale bar = 50 µm.

Eighteen HNSCC tumour specimens were collected for PDX engraftment into NSG and NOD scid mice from patients undergoing surgery in Auckland City Hospital between December 2014 and November 2018. Eleven of the tumour specimens grew as first generation (P1) PDX tumours in mice, for a take rate of 61%. These were subsequently passaged into P2 mice for characterisation and P3 mice for evofosfamide therapy studies. Ten PDX models showed good growth characteristics as P2 and P3 tumours. These PDX tumours were derived from clinical specimens from a range of sites within the head and neck region at a moderate or advanced stage and were either primary or recurrent (Table 1). One of the ten patient specimens was p16-positive and therefore likely to be HPV-positive. The histopathology of all ten tumour models closely resembled clinical HNSCC disease with cellular heterogeneity and abundant regions of keratinous tumour cells (Figure 1; Figure S1).

### 3.2. Anticancer Efficacy of Evofosfamide

**Figure 2:**
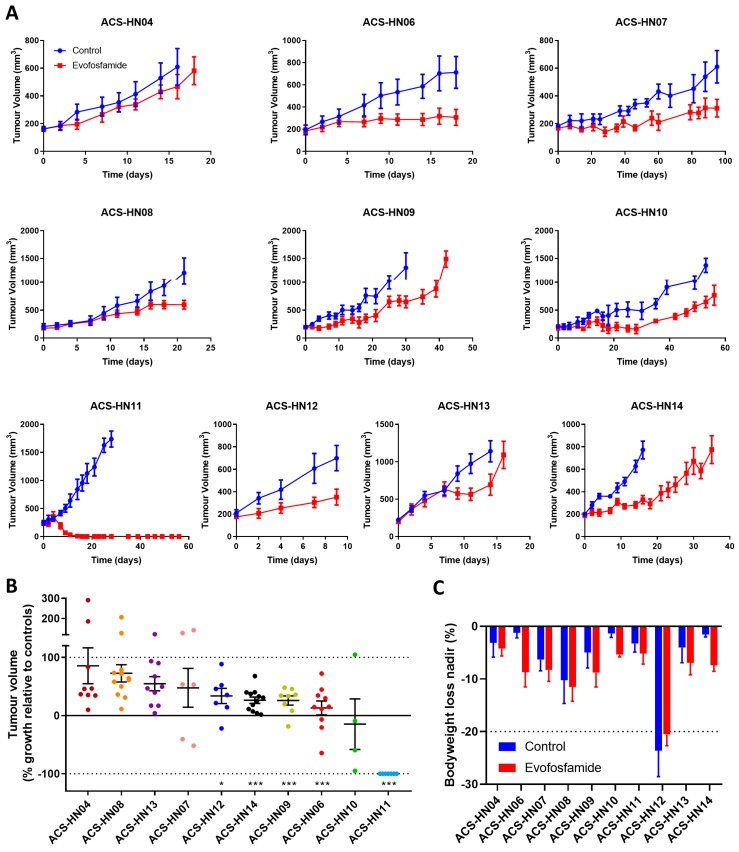
Anticancer efficacy of evofosfamide in ten head and neck squamous cell carcinoma (HNSCC) PDX models. NOD scid or NIH-III mice with P3 PDX tumours were treated with 50 mg/kg evofosfamide by IP injection qd × 5 for three weeks or control vehicle. (**A**) Average tumour growth. (**B**) Waterfall plot of tumour growth in evofosfamide-treated mice presented as change in tumour volume from starting size relative to the average control value at the end of treatment (or endpoint if earlier). The dotted line at 100% represents equal growth to controls, the dotted line at −100% represents complete regression and the line at 0% represents starting tumour size prior to treatment. * *P* < 0.05; *** *P* < 0.001 compared to controls by student’s T-test. (**C**) Bodyweight loss nadir in mice over the duration of the study. The dotted line at −20% indicates weightloss endpoint. Data in each plot represent either individual values or the mean ± SEM for *n* = 7–12 tumours (except ACS-HN10: *n* = 3–4).

The anticancer efficacy of evofosfamide as a single agent was determined in all ten PDX models to evaluate its ability to inhibit HNSCC tumour growth when administered at a multiple dose schedule of 50 mg/kg qd × 5 for three weeks. Evofosfamide had variable single-agent anticancer activity across the ten tumour models with the greatest activity observed in the p16-positive PDX model ACS-HN11, where all eight mice treated with evofosfamide had no tumours remaining within two weeks of treatment, while minimal single-agent activity was observed in other models (e.g., ACS-HN04; Figure 2A). Waterfall plots were generated by comparing each treated tumour to the control average for that tumour model at the completion of treatment (day 18) or the time point prior where a full dataset was available (day 9, ACS-HN12; day 14, ACS-HN13; day 16, ACS-HN04, ACS-HN07 and ACS-HN14). Evofosfamide significantly delayed tumour growth compared to controls in five PDX models (ACS-HN06, ACS-HN09, ACS-HN11, ACS-HN12 and ACS-HN14) by the end of treatment (Figure 2B; Figure S2). Additionally, tumours were allowed to grow beyond treatment completion until they reached 4× starting size (RTV^4^). The median time for tumours to reach RTV^4^ was significantly prolonged compared to controls in five PDX models (ACS-HN06, ACS-HN09, ACS-HN10, ACS-HN11, and ACS-HN14; Figure S3). Evofosfamide was well tolerated in all but two PDX models, with no clinical signs of toxicity and no or minor loss of bodyweight relative to controls (Figure 2C). Considerable weight loss was observed in the ACS-HN12 PDX model regardless of treatment, requiring most animals to be culled prior to study endpoint, with only 4/10 control tumours and 2/9 treated tumours reaching RTV^4^ for this model. ACS-HN07 was the only tumour model grown in NIH-III mice. The tumours grew very slowly and two mice (one control, one treatment) had to be culled due to abdominal bleeding; therefore, NOD scid mice were the preferred host for subsequent efficacy studies.

### 3.3. Hypoxia Status of PDX Models

**Figure 3:**
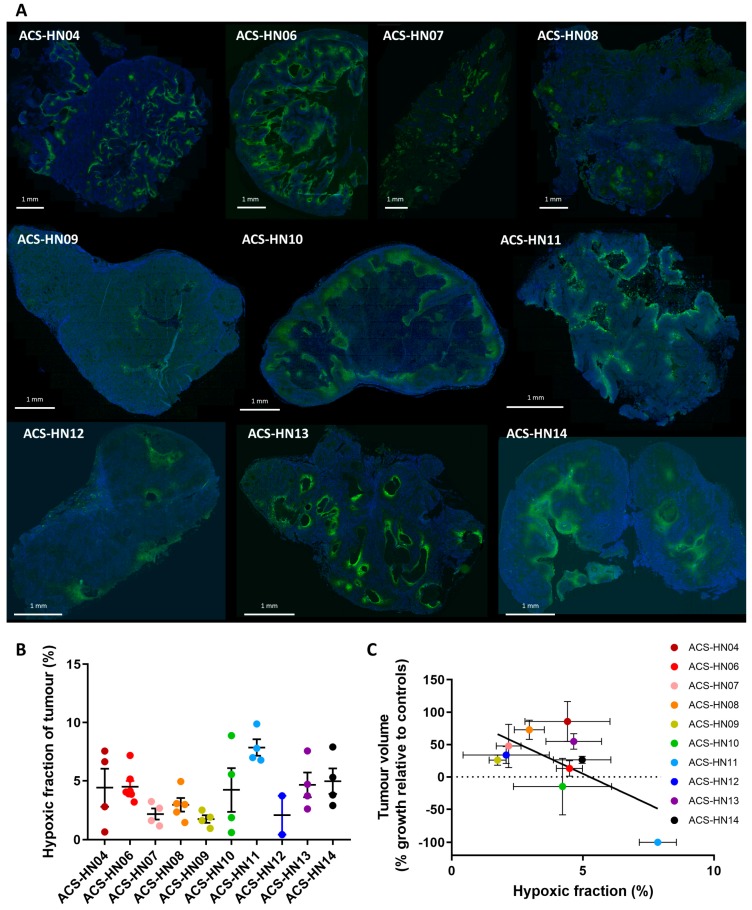
Hypoxic fraction of HNSCC PDX models by pimonidazole immunohistochemistry. (**A**) Representative whole tumour immunostains for each PDX model. Green, pimonidazole; blue, Hoechst 33258. (**B**) Hypoxic fraction determined as the pimonidazole-positive fraction of the viable tumour. Symbols represent individual tumours and mean ± SEM for *n* = 2–7 tumours. Data for ACS-HN06, ACS-HN07, and ACS-HN08 comes from [49]. (**C**) Comparison of evofosfamide antitumour efficacy (tumour growth in evofosfamide-treated mice relative to controls) to the pimonidazole-positive hypoxic fraction. Data represent mean ± SEM for *n* = 2–12 tumours.

The hypoxia status of the ten PDX models was determined by pimonidazole immunohistochemistry to determine if the tumour models that were most sensitive to evofosfamide were highly hypoxic. The presence of pimonidazole adducts as a hypoxia marker was observed in all PDX tumours (Figure 3A), frequently surrounding necrotic (absence of Hoechst 33258 nuclear staining) or keratinous areas as confirmed by examination after haematoxylin and eosin staining, although the extent of staining appeared greater in some models than others. Quantitation of the hypoxic fraction revealed this variability in staining, with hypoxic fraction values ranging from 7.9 ± 0.7% (ACS-HN11) to 1.7 ± 0.4% (ACS-HN09) (Figure 3B). Inter-tumour variation in hypoxic fraction was also present for some models. The quantitated hypoxic fraction for each of the ten PDX models was compared to the average change in tumour growth induced by evofosfamide treatment, revealing a weak but significant correlation between pimonidazole staining and evofosfamide anticancer efficacy across the HNSCC PDX models (*r* = −0.652, *P* = 0.04; Figure 3C).

**Figure 4:**
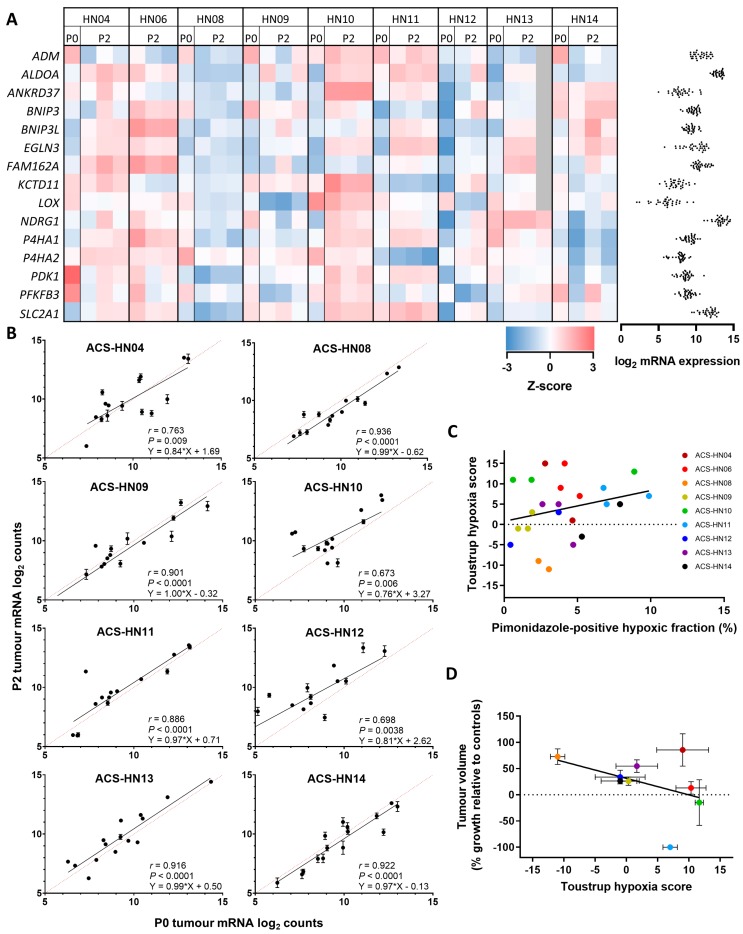
Hypoxia status of HNSCC PDX tumours by Toustrup hypoxia gene signature analysed by NanoString. (**A**) Heatmap of mRNA expression of 15 Toustrup signature genes in P0 and P2 PDX tumours determined as z-score relative to other PDX tumour samples. Red indicates high expression, blue indicates low expression, grey indicates not determined. Gene expression determined as log_2_ counts normalised to positive control and three housekeeping genes. Individual counts shown on the right. (**B**) Comparison of mRNA expression of 15 Toustrup genes in P0 (*n* = 1) and P2 (*n* = 2–3) PDX tumours. Symbols represent mean ± SEM. Dotted lines represent equal expression. (**C**) Comparison of hypoxic fraction assessed by Toustrup hypoxia score and pimonidazole staining. Symbols represent individual tumours. (**D**) Comparison between evofosfamide anticancer efficacy (tumour growth in evofosfamide-treated mice relative to controls at end of treatment or endpoint) and Toustrup hypoxia score. Symbols represent mean ± SEM for *n* = 2–12 tumours.

An orthogonal approach was used to determine the hypoxia status of the PDX tumours, by evaluating the mRNA expression of genes known to be regulated by hypoxia in HNSCC, using the Toustrup hypoxia gene signature. The mRNA expression of the 15 Toustrup hypoxia signature genes was determined by NanoString in nine PDX models, revealing differences in expression of hypoxia signature genes between different models, and in some instances (particularly ACS-HN04) between tumours of the same PDX model (Figure 4A). Toustrup gene expression was also determined in the clinical specimens (P0 tumours) for eight of the nine PDX models where P0 tissue was available. A significant correlation in Toustrup gene expression was observed between P0 and P2 tumours in all tumour models, with five models in particular showing strong correlations (*r* values > 0.85 and log-log slopes between 0.97 and 1.00) (Figure 4B). In a manner analogous to analysis of other hypoxia gene signatures [57,58], a Toustrup score was generated for each tumour based on whether the expression of each individual gene was above or below the median expression across all PDX tumours. Comparison of each tumour’s Toustrup score to its hypoxic fraction as determined by pimonidazole staining, revealed no correlation (*r* = 0.272, *P* = 0.21, Figure 4C). Similarly, there was no correlation between average Toustrup score and evofosfamide antitumour efficacy across the nine PDX models (*r* = −0.415, *P* = 0.27, Figure 4D).

### 3.4. MKI67, POR and SLFN11 Expression in PDX Tumours

**Figure 5:**
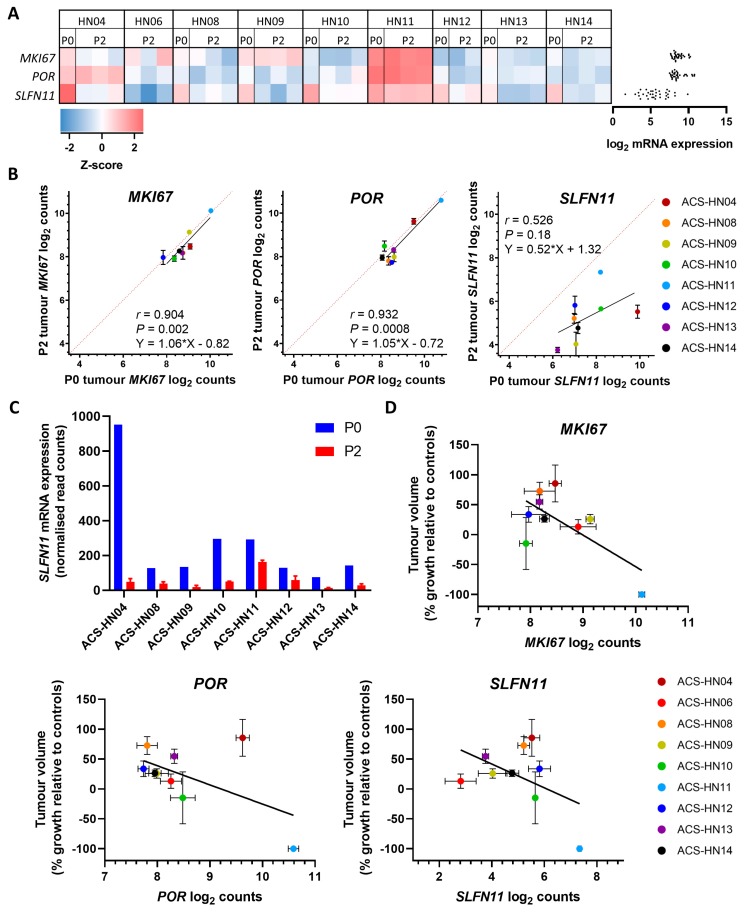
Expression of *MKI67*, *POR*, and *SLFN11* in HNSCC PDX models. (**A**) Heatmap of mRNA expression of *MKI67*, *POR*, and *SLFN11* genes in P0 and P2 PDX tumours determined as z-score relative to other PDX tumour samples. Red indicates high expression, blue indicates low expression. Gene expression determined as log_2_ counts normalised to positive control and three housekeeping genes. Individual counts shown on right. (**B**) Comparison of *MKI67*, *POR* and *SLFN11* mRNA expression in P0 (*n* = 1) and P2 (*n* = 2–3) tumours across eight PDX models. Symbols represent mean ± SEM. Dotted lines represent equal expression. (**C**) *SLFN11* expression in P0 and P2 tumours for eight PDX models. Bars represent individual values (P0) or the mean ± SEM for *n* = 2–3 tumours (P2). (**D**) Comparison between evofosfamide anticancer efficacy (tumour growth in evofosfamide-treated mice relative to controls at end of treatment or endpoint) and *MKI67*, *POR* and *SLFN11* expression in HNSCC PDX models. Symbols represent mean ± SEM for *n* = 2–12 tumours.

Next, we investigated three candidate evofosfamide sensitivity genes in our PDX models: the one electron reductase *POR* [59] that can convert evofosfamide into its active form [53], the endoribonuclease *SLFN11*, which plays a major role in sensitivity to DNA damaging agents [60,61,62], and a marker of proliferation, *MKI67*. There was moderate expression of *MKI67* and *POR* across the PDX tumour models, but low expression of *SLFN11* (Figure 5A). Interestingly, there was a significant correlation for *MKI67* (*r* = 0.904, *P* = 0.002) and *POR* (*r* = 0.932, *P* = 0.0008) expression between P0 and P2 tumours across the different tumour models, but a poor correlation for *SLFN11* expression (*r* = 0.526, *P* = 0.18), with P2 tumours from all nine PDX models having lower *SLFN11* expression than their corresponding P0 tumours (Figure 5B). Across all PDX models, there was a 5.2-fold reduction in *SLFN11* expression in P2 tumours vs. P0 tumours, with the greatest difference (a 19.8-fold reduction) observed in ACS-HN04 (Figure 5C). There was a weak but significant correlation between evofosfamide efficacy and *MKI67* expression (*r* = −0.682, *P* = 0.04; Figure 5D), but not between evofosfamide efficacy and *POR* (*r* = −0.558, *P* = 0.12) or *SLFN11* (*r* = −0.481, *P* = 0.19), with the highest expression for each gene observed in ACS-HN11, the tumour model that was most sensitive to evofosfamide.

## 4. Discussion

We established ten PDX models of HNSCC that had suitable growth characteristics for passaging into second and third generation cohorts of mice for characterisation and evaluation of drug sensitivity, respectively. Our PDX models showed histopathology characteristic of HNSCC that closely matched the original tumours they were derived from. Furthermore, to the best of our knowledge, we have shown for the first time a close correlation in the expression of a hypoxia gene signature between PDX tumours and surgical specimens, while the pimonidazole-positive hypoxic fraction was in a similar range to that reported for clinical HNSCC tumours [63,64,65], in contrast to some cell line xenograft models which can over-predict clinical tumour hypoxia and thus over-predict HAP activity [11]. Combined, these data support our PDX models as appropriate models of HNSCC for evaluating the efficacy of anticancer agents whose activity is influenced by the hypoxic tumour microenvironment, such as the HAP evofosfamide.

Since tumour hypoxia promotes poor prognosis and failure of chemoradiotherapy in HNSCC, targeting of hypoxia provides a therapeutic opportunity in this setting. Expanding on our earlier findings [49], we found that evofosfamide had single-agent antitumour activity in several clinically-relevant PDX models of HNSCC, providing further evidence for clinical testing of evofosfamide in this setting. Evofosfamide has been evaluated in 27 completed or ongoing clinical trials, but not yet in HNSCC, other than in five patients in a phase II expansion cohort to the initial phase I trial (NCT00495144), all of whom showed disease control (two with confirmed partial responses, three with stable disease) [49]. However, it has become evident that for HAPs such as evofosfamide to be successful in the clinic they need to be co-developed with additional biomarker support to identify the tumours most likely to be sensitive to HAP therapy [50,66].

To that end, we investigated whether tumour hypoxia status could determine sensitivity to evofosfamide in our HNSCC PDX models and act as a predictive biomarker. Despite hypoxia being essential for the activation of evofosfamide [49,52,53,67], we found that tumour hypoxia status, determined by both pimonidazole staining and by the Toustrup gene signature, had at best a weak correlation with the antitumour efficacy of evofosfamide in our HNSCC models. This suggests that using hypoxia for patient selection, as has been proposed [50,66], may not be sufficient on its own for predicting evofosfamide activity and that additional biomarkers may be required, consistent with the complex mechanism of action of this prodrug. However, it should be noted that there was not a large range in variation in the antitumour efficacy of evofosfamide nor the pimonidazole-positive hypoxic fraction across our PDX models, which reduced the statistical power to identify positive correlations. Furthermore, the contribution of the murine microenvironment was not specifically investigated in this study and whether or not it could influence hypoxic fraction or evofosfamide sensitivity. Therefore, although the data suggest it is unlikely that hypoxia alone determines sensitivity to evofosfamide and that other factors also contribute, a larger number of models with wider variance in tumour hypoxia and evofosfamide sensitivity and with defined murine microenvironment contributions is required in order to robustly assess the performance of tumour hypoxia on evofosfamide activity.

Unexpectedly, there was a lack of correlation between the pimonidazole-positive hypoxic fraction and the Toustrup hypoxia score of the PDX tumours. This may be due to the Toustrup genes being largely HIF-target genes that are upregulated under different conditions than required for the formation of pimonidazole protein adducts which require one-electron reductase enzymes to generate active hydroxylamine intermediates [68,69]. In addition, binding of 2-nitroimidazole probes such as pimonidazole requires more extreme hypoxia than does stabilisation of HIF-1 [11], which may also contribute to the discordance between the two endpoints. Alternatively, the lack of correlation may reflect the low variability in hypoxic fraction between models or experimental differences between the two assays; for instance, hypoxic cells within necrotic areas contributed to gene expression profiles but were excluded from pimonidazole quantitation. ACS-HN06 and ACS-HN10 tumours, which had high Toustrup scores but moderate to low pimonidazole-positive hypoxic fractions, were highly necrotic. Furthermore, Toustrup gene expression was determined from a small sample from each tumour that may not reflect the average hypoxic fraction of the tumour, due to intratumoural heterogeneity [70], nor may it reflect the average gene expression across the tumour, due to the heterogeneous expression of hypoxia-related genes in HNSCC [71]. Additionally, with no reference dataset available, the Toustrup scores are relative across tumours from the nine PDX models, meaning that those with the highest score are not necessarily highly hypoxic but rather more hypoxic than the other PDX tumours, while those with the lowest score are not necessarily mildly hypoxic, but rather less hypoxic than the other PDX tumours. Activation of evofosfamide can occur under mild hypoxia, which makes an important contribution to the drug’s antitumour efficacy because of its lack of a bystander effect [52,72] and this may help explain why it was still active in tumour models with low levels of hypoxia (e.g., ACS-HN09).

We evaluated *MKI67*, *POR* and *SLFN11* expression as other potential candidate predictive biomarkers of evofosfamide sensitivity [49,53,54]. Of these genes, only *MKI67* showed evidence of a correlation with the antitumour efficacy of evofosfamide, consistent with our previous finding that evofosfamide is more potent in highly proliferative HNSCC cell lines [49]. However, this result should be viewed with caution due to the large dependence of this correlation on the ACS-HN11 tumour model. Evofosfamide showed striking antitumour activity in ACS-HN11 with all tumours showing complete regression within two weeks of treatment. The ACS-HN11 tumour model had the highest pimonidazole-positive hypoxic fraction as well as the highest *MKI67*, *POR* and *SLFN11* expression, but was also p16-positive, thus an ~85–90% likelihood of being HPV-positive, and originated from a tumour that was recurrent post chemoradiotherapy. As the only p16-positive tumour model in our collection, it is not clear whether the hypoxia or genetic biomarkers individually or collectively contributed to the notable sensitivity of this model, or whether this sensitivity reflects a different feature of HPV-positive HNSCC (which is known to be chemosensitive relative to HPV-negative disease [73]) or of recurrent tumours, since evofosfamide was also active in recurrent ACS-HN06 and ACS-HN10 tumours. Further testing is therefore required in additional HPV-positive and recurrent HNSCC tumour models to confirm this sensitivity of evofosfamide.

*SLFN11* expression was reduced in all P2 PDX models relative to the original P0 clinical specimen. *SLFN11* encodes an endoribonuclease that regulates DNA repair. It is epigenetically regulated by methylation, with hypermethylation likely responsible for the decreased expression in PDX tumours, as seen previously in cancer cell lines [74,75]. Since *SLFN11* promotes sensitivity to DNA damaging agents that interfere with DNA replication fork progression [60,61,62], possibly including evofosfamide [54], reduced expression of *SLFN11* in PDX tumours relative to clinical tumours suggests the PDX tumours could potentially under-predict the clinical activity of evofosfamide in HNSCC.

In summary, we have established 10 PDX models of HNSCC with similar histology and a close correlation in mRNA expression of the Toustrup hypoxia gene signature to the clinical tumours they were derived from, providing evidence that they are useful models for examining the anticancer efficacy of therapies that are influenced by tumour hypoxia. Using these clinically-relevant PDX models, we have demonstrated antitumour efficacy of evofosfamide in HNSCC, which supports clinical evaluation of the drug in this setting. Our data suggest that evofosfamide sensitivity cannot be robustly predicted by hypoxia, *MKI67*, *POR*, or *SLFN11* expression on their own, but rather is likely to be multifactorial, incorporating tumour hypoxia and other genes that influence the activation and mechanism of action of evofosfamide. However, a larger number of models with wider variance in biomarker expression and evofosfamide sensitivity is required in order to confirm these findings and to identify predictive biomarkers in support of precision medicine therapy of evofosfamide in HNSCC.

## Figures and Tables

**Table 1:** Characteristics of patient-derived xenograft (PDX) models.

Model	Site	Derivation	Stage	p16 Status
ACS-HN04	Tongue	Primary	pT2 N1 Mx	Negative
ACS-HN06	Oropharynx	Recurrent	T4a N2b M0	Negative
ACS-HN07	Tongue	Recurrent	T3 N0 M0	Negative
ACS-HN08	Tongue	Primary	pT4a N2b M0	Negative
ACS-HN09	Tongue	Primary	pT2 N2b M0	Negative
ACS-HN10	Maxilla	Recurrent	T2 N0 M0	Negative
ACS-HN11	Tongue	Recurrent	T2 N0 M0	Positive
ACS-HN12	Tongue	Primary	pT2 N0 Mx	Negative
ACS-HN13	Hypopharynx pyriform sinus	Primary	pT4a N3b M0	Negative
ACS-HN14	Buccal mucosa	Primary	pT4a N2 Mx	Negative

## Supplementary Materials

The following are available online at https://www.mdpi.com/2073-4409/8/7/717/s1, Figure S1: Histopathology of ten PDX models at P0; Figure S2: Tumour growth in control mice relative to the average control value at the end of treatment (or endpoint if earlier); Figure S3: Antitumour efficacy of evofosfamide in ten HNSCC PDX models determined as the time taken for tumours to quadruple in size (RTV^4^, relative tumour volume × 4). Table S1: Oligonucleotide probes for NanoString gene analysis.
