# Supplementary material for: Impact of Tumour Hypoxia on Evofosfamide Sensitivity in Head and Neck Squamous Cell Carcinoma Patient-Derived Xenograft Models
Source: Cells. 2019 Jul 13;8(7):717. doi: 10.3390/cells8070717 (PMC6678517; doi:10.3390/cells8070717)
Supplement: Supplementary file 1 [file cells-08-00717-s001.pdf]

cells

Article

Impact of Tumour Hypoxia on Evofosfamide Sensitivity in Head and Neck Squamous Cell Carcinoma Patient-Derived Xenograft Models

Julia K. Harms [1], Tet-Woo Lee [1,2], Tao Wang [1], Amy Lai [1,3], Dennis Kee [4], John M. Chaplin [5], Nick P. McIvor [5], Francis W. Hunter [1,2], Andrew M. J. Macann [6], William R. Wilson [1,2] and Stephen M.F. Jamieson [1,2,3,*]

1 Auckland Cancer Society Research Centre, University of Auckland, Auckland 1023, New Zealand
2 Maurice Wilkins Centre for Molecular Biodiscovery, University of Auckland, Auckland 1010, New Zealand
3 Department of Pharmacology and Clinical Pharmacology, University of Auckland, Auckland 1023, New Zealand
4 LabPLUS, Auckland City Hospital, Auckland 1023, New Zealand
5 Department of Otolaryngology–Head and Neck Surgery, Auckland City Hospital, Auckland 1023, New Zealand
6 Department of Radiation Oncology, Auckland City Hospital, Auckland 1023, New Zealand
* Correspondence: s.jamieson@auckland.ac.nz

Supplement:

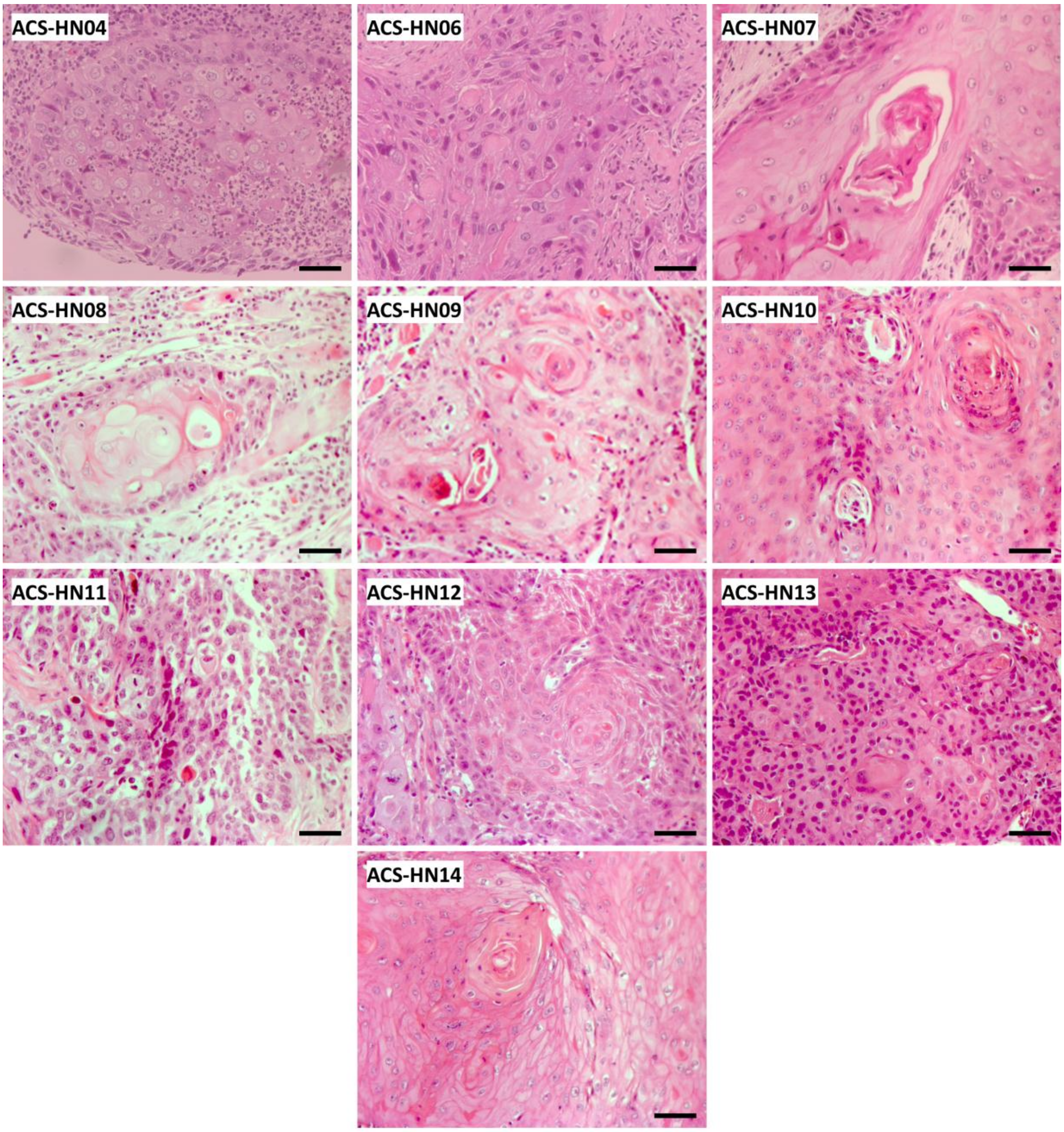

Figure S1. Histopathology of ten PDX models at P0. Scale bar = 50 μm.

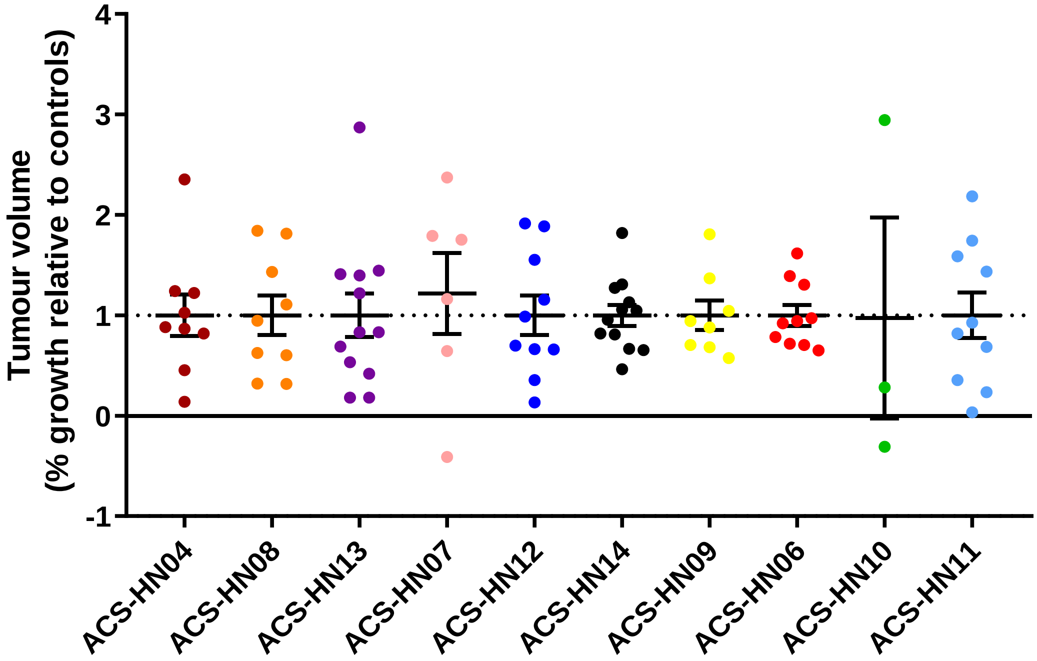

Figure S2. Tumour growth in control mice relative to the average control value at the end of treatment (or endpoint if earlier). Data represents individual values and the mean ± SEM.

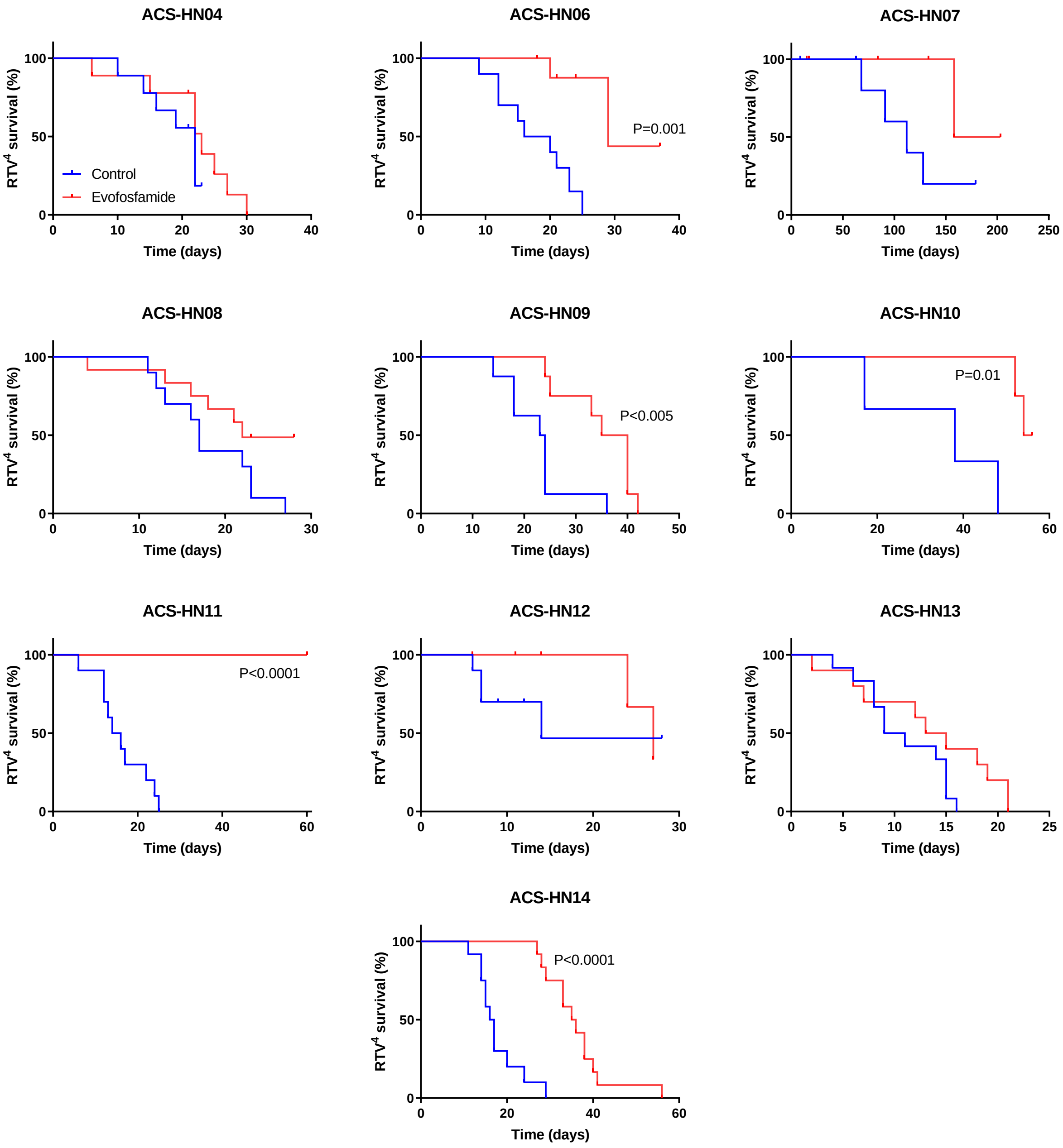

Figure S3. Antitumour efficacy of evofosfamide in ten HNSCC PDX models determined as the time taken for tumours to quadruple in size (RTV^4, relative tumour volume × 4). NOD scid mice with P3 PDX tumours were treated with 50 mg/kg evofosfamide by IP injection qd × 5 for three weeks or control vehicle. Plots for ACS-HN06, ACS-HN07 and ACS-HN08 reproduced with permission from [49] © (2018) American Society for Clinical Investigation.

Table S1. Oligonucleotide probes for NanoString gene analysis.

Gene	Accession	Probes
RPS13	NM_001017.2	GAATCAGACGGAATTTAGCATCCTTATCCTTTCTGTTCCTCTCAAGATGCCCTCAAGACCTAAGCGACAGCGTGACCTTGTTTCA<br>CGAAAGCCATGACCTCCGATCACTCTCGCTTGGTCTTATAATATCGAGCCAAACGGTGAATCCGGCTCTCTATTA
RPLP0	NM_001002.3	CTGCATCTGCTTGGAGCCCACATTGTCTGCTCCCACAATGAAACATTTCGCATCCTCTTCTTTTCTTGGTGTTGAGAAGATGCTC<br>CGAAAGCCATGACCTCCGATCACTCCACCACAGCCTTCCCGCGAAGGGACATGCGGATCTG
OAZ1	NM_004152.2	AATAAGTTAGCTGAAAGATTGTGATCCCTCTGACTATTCCCTCGCCCACCCACAATTCTGCGGGTTAGCAGGAAGGTTAGGGAAC<br>CGAAAGCCATGACCTCCGATCACTCTGTCGTTGGACGTTAGTTCCTCTGTTACATTCAGCCGATCATCGGAGTAG
ADM	NM_001124.1	TGCACACAAACACACTCACATTCCACGCGGCGAACAACTTTACACCTCGCCTGTTGAGATTATTGAGCTTCATCATGACCAGAAG<br>CGAAAGCCATGACCTCCGATCACTCACTCGGTGTTTCCTTCTTCCACACAGGAGGTAATCAGTCTTTCTCTTTCA
ALDOA	NM_184041.2	CACCACACACCACTGTCACGAGGGAAGAAAGAGCGCGGGCAAGCCAGCAAAGACGCCTATCTTCCAGTTTGATCGGGAAACT<br>CGAAAGCCATGACCTCCGATCACTCTTATTTGGCAGTGTGCCGGAAAGGGTGATGGACTTAGCATTCACAGACGA
ANKRD37	NM_181726.2	TCCAGGCTTCCAACTTTTGCTGCCTTGTGTAGTGGCGAACCTAACTCCTCGCTACATTCCTATTGTTTTC<br>CGAAAGCCATGACCTCCGATCACTCTATTACATAAATCAATTTGGGCATCACTGGCTACAAGCAGGCTTAGGCAC
BNIP3	NM_004052.3	CTGCGGGATGTGCTTCAGCTGCGGGCGGTGGGAAAGCCAATTTGGTTTTACTCCCCTCGATTATGCGGAGT<br>CGAAAGCCATGACCTCCGATCACTCAGAGGGCAACTGCGGCGATCGGAGTCCGCGCCGGG
BNIP3L	NM_004331.2	GGATGGTACGTGTTCCAGCCCCCCATTTTTCCCATTGCCATTATCATTGCCTTTCGGGTTATATCTATCATTTACTTGACACCCT<br>CGAAAGCCATGACCTCCGATCACTCTGTGCATCCAAAAGAATCTTCTCCATGTCTCCATTGTGGATGGAGGATGA
EGLN3	NM_022073.3	ATGAATGATTTCCCCTCTGGAAATATCCGCAGGATCCCACCATGTAGCTTCAACAGCCACTTTTTTTCCAAATTTTGCAAGAGCC<br>CGAAAGCCATGACCTCCGATCACTCGATCTGACCAGAAGAACAGGAGTCTGTCAAAAATGGGCTCCACATCTGCT
FAM162A	NM_014367.3	AAGCGACCTGACCATATGAGGATCTTTTTCTGCCAATCCGTAGGTTTGTGCACCGTGTGGACGGCAACTCAGAGATAACGCATAT<br>CGAAAGCCATGACCTCCGATCACTCCATCAAGCATCTCCAACGAGACAGTCTCTGGGATTTCATCTTCCTTTTTG
KCTD11	NM_001002914.2	TCTGAAGGGATGGCCTCCGAGACCCCGGGCAGATCCCTGGAGTTTATGTATTGCCAACGAGTTTGTCTTT<br>CGAAAGCCATGACCTCCGATCACTCAACCAGCAGGTGACGGTGGCGAGCACAGGGCCGAC
LOX	NM_002317.4	TACGGTGAAATTGTGCAGCCTGAGGCATACGCATGATGTCCTGTGTAGCAGATAAGGTTGTTATTGTGGAGGATGTTACTACA<br>CGAAAGCCATGACCTCCGATCACTCAGAACACCAGGCACTGATTTATCCATTGGGAGTTTTGCTTTGCCTTCTAA
NDRG1	NM_001135242.1	ACACAACAACAAGGAATCCCTTACATCGAGTAACCCCAATTCCACCCCCACTGTTGAGATTATTGAGCTTCATCATGACCAGAAG<br>CGAAAGCCATGACCTCCGATCACTCTCCTGGAACCAAGCTGGGATCCACAGAAATCACAACTGCACTGGATCTCA
P4HA1	NM_000917.3	CAGAGACTTTATCTATGGTAGAAATCTCGCCTTCATCCAGTTGCCTTAGGCAAAGACGCCTATCTTCCAGTTTGATCGGGAAACT<br>CGAAAGCCATGACCTCCGATCACTCCTTATCCAGGTCTCCCTGCTGATATACCGCATAGCTCAAATAATCTAGAA
P4HA2	NM_004199.2	GTTAACCCTGTGATATGCTGCATCCGACGATTTACTCGGGCCACAACAGGCGAACCTAACTCCTCGCTACATTCCTATTGTTTTC<br>CGAAAGCCATGACCTCCGATCACTCGTCCTCCCACTCCATAATTTGCAACCTGTAACAATTCTGCAGTCTTTACT
PDK1	NM_002610.3	TCAGGTCTCCTTGGAAGTATTGTGCGTAAAGACGTGATATGGGCAATCCACCAATTTGGTTTTACTCCCCTCGATTATGCGGAGT<br>CGAAAGCCATGACCTCCGATCACTCCTTAATGTAGATAACTGCATCTGTCCCGTAACCCTCTAGGGAATACAGCT
PFKFB3	NM_004566.3	CATCGAAAACCGCAATTTGTCCCCCTTCTTTCGCCAGGTAGCTTTTGACACTTTCGGGTTATATCTATCATTTACTTGACACCCT<br>CGAAAGCCATGACCTCCGATCACTCTTTGGCAAAATGAAGGATCATGTGTCTCCTCTCTCTAGTAGTATTGGTGG
SLC2A1	NM_006516.2	TGAGTGGTTGGGTAGGAAGAGATGGGAAGGGGCAAATCCTAATGGAGCCTCAACAGCCACTTTTTTTCCAAATTTTGCAAGAGCC<br>CGAAAGCCATGACCTCCGATCACTCTCCCTGCACTCCAGTGCTCCCAACTGGTCTCAGGTAAAGAAAGATTAATT
MKI67	NM_002417.2	CTGATGGCATTAGATTCCTGCACGCTAAGAGTTCTCCCTCTACATCTGCACCGTGTGGACGGCAACTCAGAGATAACGCATAT<br>CGAAAGCCATGACCTCCGATCACTCGTCTTTCTCTTCACCTACTGATGGTTTAGGCGTGTGCATGGCTTTGCCTG
POR	NM_000941.2	TGCTGAAAAGAGATACTTCTTCGGCCACCGCCTCGGACACGGTGGAGCCTGGAGTTTATGTATTGCCAACGAGTTTGTCTTT<br>CGAAAGCCATGACCTCCGATCACTCCCAGTAGGTTAGGAGACCCACGATGAGCGAAAACAGAATCATGTCCGTCA
SLFN11	NM_001104587.1	AATGCCTCTCTTGAGTCCATGGAACGCACAGAGGTCTCAGATCTACGGTACAGATAAGGTTGTTATTGTGGAGGATGTTACTACA<br>CGAAAGCCATGACCTCCGATCACTCAAGGTCCTTCTTCCAAGATTTTTGGCTTCCTTTTGGTCTTCAGGAAACAG
